# Selenium-Mediated Synthesis of Tetrasubstituted Naphthalenes through Rearrangement

**DOI:** 10.3390/molecules200610866

**Published:** 2015-06-12

**Authors:** James Tancock, Thomas Wirth

**Affiliations:** School of Chemistry, Cardiff University, Cardiff CF10 3AT, UK; E-Mail: tancockJ@cardiff.ac.uk

**Keywords:** naphthols, rearrangement, selenium

## Abstract

New β-keto ester substituted stilbene derivatives have been synthesized and cyclized with selenium electrophiles in the presence of Lewis acids. This now allows access to 1,2,3,4-tetrasubstituted naphthalene derivatives as cyclization and rearrangement products.

## 1. Introduction

Organoselenium chemistry and selenium-containing reagents allow nucleophilic, electrophilic as well as radical reactions, which have been investigated intensively for the last decades. The formation of new bonds through the selenium-mediated addition to double bonds has become a key reaction for electrophilic selenium reagents and we have reported on many facets of these transformations [1,2]. Apart from heteroatom nucleophiles used in selenenylation reactions of alkenes, we have reported carboselenenylations leading to dihydronaphthalenes and benzofluorene derivatives [3]. 

## 2. Results and Discussion

**Scheme 1:**
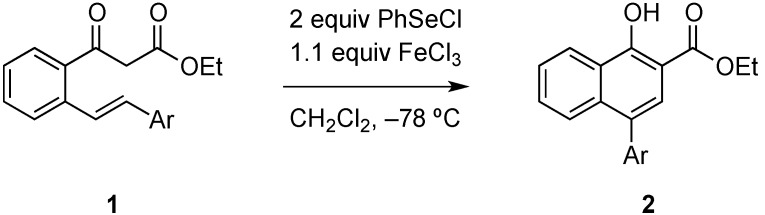
Synthesis of naphthols **2** from β-keto esters **1**.

Herein we expand on a recently reported reaction sequence of carboselenenylation and elimination, which is accompanied with a 1,2-rearrangement of an aryl group under very mild reaction conditions [4]. We had shown that the treatment of β-keto esters of type **1** with phenyl selenenylchloride in the presence of iron(III)chloride leads in high yields to the cyclization products **2**, which were the result of a sequence of carboselenenylation, 1,2-migration of the aryl substituent and elimination (Scheme 1).

There have been many natural chemicals discovered that contain a naphthalene ring in their structure. In some instances, these natural products have some biological activity. We were therefore interested to extend the above mentioned reaction sequence towards the synthesis of 1,2,3,4-tetrasubstituted naphthol derivatives. Other reactions leading to 1,2,3,4-tetrasubstituted naphthol derivatives have been described recently [5].

**Scheme 2:**
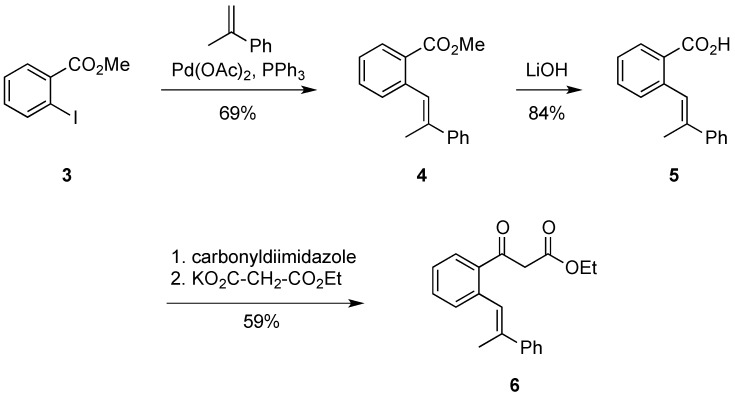
Synthesis of starting material **6**.

In order to use the reaction sequence shown in Scheme 1 for the synthesis of 1,2,3,4-tetrasubstituted naphthol derivatives, modified starting materials had to be synthesized. This was achieved by the reactions sequence shown in Scheme 2.

Firstly, the Mizoroki-Heck cross coupling of methyl 2-iodobenzoate **3** with α-methyl styrene afforded the stilbene ester **4** in good yields. The palladium-catalyzed reaction produced exclusively only one geometric isomer, as evidenced by ^1^H-NMR. The ester **4** was then hydrolyzed into its carboxylic acid derivative **5** using three equivalents of lithium hydroxide. Typically a hydrolysis of an ester using this method proceeds almost quantitatively, however in this instance 84% was the greatest yield which could be achieved. The carboxylic acid **5** was then activated using carbonyldiimidazole (CDI) in order for its conversion to the β-keto ester. After stirring the carboxylic acid **5** overnight with CDI, the transformation into stilbene **6** required the addition of potassium ethyl malonate along with MgCl_2_ and NEt_3_. After stirring overnight once more, stilbene ester **6** was isolated in an acceptable yield (59%). NMR investigations of **6** showed it was in equilibrium with its enol tautomer, this equilibrium was shifted towards the keto form.

Different selenium electrophiles have been investigated for the carboselenenylation of **6** to **7**. The different reagents and reaction conditions are shown in Table 1.

**Table 1:** Conditions for cyclization of **6** to **7**.

Entry	Reagents	Conditions	7 Yield
1	PhSeSePh, FeCl_3_	−78 °C, 1.5 h	traces
2	PhSeSePh, FeCl_3_	25 °C, 1.5 h	7%
3	PhSeOTf	−78 °C, 3 h	10%
4	PhSeCl, FeCl_3_	−78 °C, 3 h	40%
5	PhSeCl, FeCl_3_	−78 °C, 12 h	61%
6	PhSeCl, FeCl_3_	−60 → 25 °C, 5 h	96%

It is clear from these data that the diphenyl diselenide is not electrophilic enough to add across the stilbene double bond to any great extent (Table 1, entries 1 and 2). Phenylselenenyl bromide and silver triflate can be used to form the phenylselenenyl triflate *in situ*. But even this reagent did not lead to substantial amounts of the cyclization product. Only the reagent combination of phenylselenenyl chloride and iron(III) chloride could be optimized successfully and finally provided product **7** in almost quantitative yield (Table 1, entry 6).

**Scheme 3:**
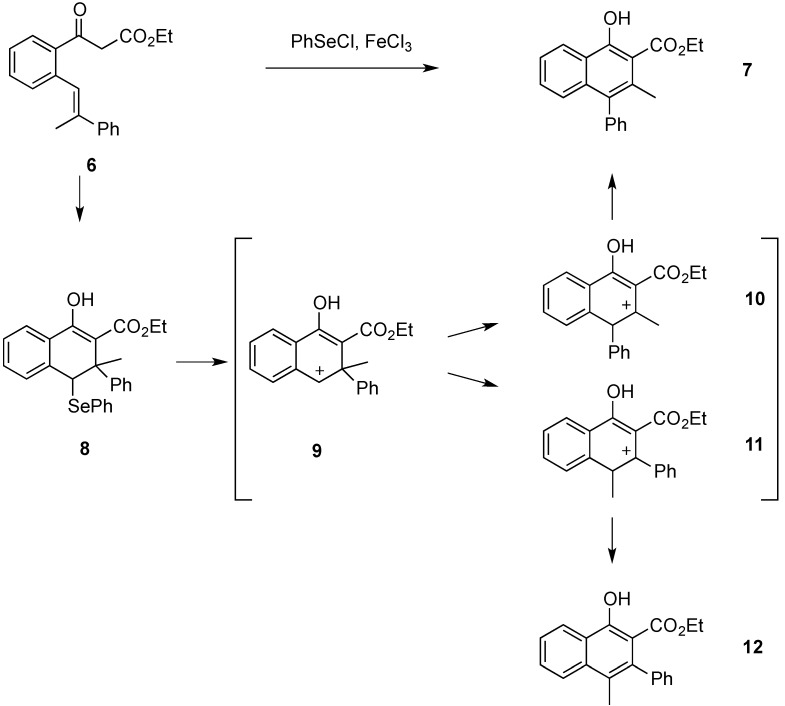
Cyclization and rearrangement of β-ketoester **6** to naphthol derivative **7**.

Attempts were also made to isolate dihydronaphthalene **8** by carrying out the carbocyclization procedure at −78 °C and using only one equivalent of phenylselenenyl chloride. However, an isolation of **8** via preparative TLC was not successful. The phenylselenenyl and the phenyl substituent in **8** are *trans*-oriented due to the *E-*double bond geometry of **6**. We have already established such a relationship in related reactions where starting materials with a *Z*-double bond configuration would not react due to the steric interactions in the cyclization intermediate [6]. As already stated, when carrying out the carbocyclization of stilbene **6**, naphthol derivative **7** was obtained as the only product as shown in Scheme 3. Compound **12** was not observed. Therefore, the migration of the phenyl ring is the exclusive process occurring during the reaction. In order to quantify this, the relative energies of carbocations **9**, **10**, and **11** were calculated. The calculations were performed using the Gaussian 03 software package [7]. Geometries were optimized separately for all structures and the energies were calculated using the B3LYP/6-31G (d) level of theory. Solvent effects were considered using the PCM solvent model available through Gaussian. Intermediates **10** and **11** were both more stable than **9**, by 17.37 and 18.96 kcal∙mol^−1^, respectively. Intermediate **10** had a net stabilization of 1.59 kcal∙mol^−1^ over **11**. Considering the carbocations **10** and **11** in their protonated ketone resonance forms, the calculations on those resonance structures led to the same conclusion. The resonance structure of **10** was 1.50 kcal∙mol^−1^ lower in energy that that of **11**. These protonated ketone forms may be a better representation of the intermediates because usual logic would predict **11** being the more stable of the two with the positive charge located in a benzylic position. The phenyl substituent could also stabilize the positive charge through the formation of a phenonium intermediate. We had proposed such intermediates in previous rearrangements [8], but in this case the calculation of a phenonium ion intermediate was unsuccessful.

**Figure 1:**
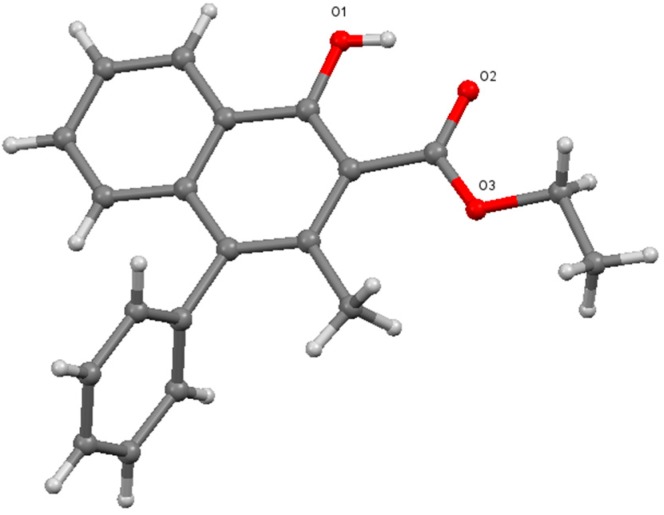
Single crystal X-ray structure of **7** [9].

The structural analysis of **7** was carried out to determine the position of both, the phenyl and methyl groups. ^1^H- and ^13^C-NMR spectra are in agreement with the literature values [5]. High resolution mass data also support the formation of the cyclic product. However, to unambiguously determine the positions of the substituents, it was required to analyze the crystals of **7** using single crystal X-ray diffraction. As it can be seen below (Figure 1), this analysis proved that the phenyl group migrated to the 4 position instead of the methyl group, in line with the assumption made according to the previous findings.

## 3. Experimental Section 

*Methyl (E)-2-(2-phenylprop-1-en-1-yl)benzoate *(**4**) [10]. A mixture of methyl 2-iodobenzoate (15 mmol, 4.0 g), α-methylstyrene (18 mmol, 2.3 mL), triethylamine (32 mmol, 4.4 mL), palladium acetate (0.48 mmol, 323 mg) and triphenylphosphine (0.96 mmol, 251 mg) were added to a round bottom flask and heated under reflux at 120 °C for 5 h. After cooling, 10% aq. HCl (100 mL) was added slowly with stirring. The resulting mixture was extracted with ethyl acetate. The organic layers were collected and concentrated under vacuum. The crude product was purified by column chromatography (EtOAc:hexane, 1:12) to give the product **4** as yellow oil (2.61 g, 10.35 mmol) in 69% yield. ^1^H-NMR (300 MHz, CDCl_3_): δ = 8.00 (d, *J* = 7.9 Hz, 2H), 7.81 (dd, *J* = 7.8, 1.7 Hz, 1H), 7.63–7.58 (m, 2H), 7.40–7.35 (m, 3H), 7.33–7.29 (m, 2H), 3.94 (s, 3H), 2.11 (d, *J* = 1.3 Hz, 3H) ppm.

*(E)-2-(2-Phenylprop-1-en-1-yl)benzoic acid* (**5**) [11]. Stilbene **4** (12.5 mmol, 3.0 g) was dissolved in a 90 mL solution of THF:MeOH:H_2_O (4:1:1). LiOH was added at room temperature with stirring. The reaction was heated for 12 hours at 70 °C. The reaction was cooled with ice and neutralized with a 1 M HCl solution. The solvents were removed under vacuum and the organic products taken up in EtOAc, then washed with water and brine. The crude product was then recrystallized in ethanol to give the clean product **5** as colorless crystals in 84% yield (10.5 mmol, 2.49 g). ^1^H-NMR (300 MHz, CDCl_3_): δ = 8.06 (d, *J* = 8.0 Hz, 2H), 8.01 (dd, *J* = 7.8, 1.6 Hz, 1H), 7.62–7.57 (m, 2H), 7.44–7.37 (m, 3H), 7.32 (d, *J* = 2.8 Hz, 2H), 2.13 (d, *J* = 1.3 Hz, 3H) ppm. HRMS (NSI): *m/z* [M – H] calcd. for C_16_H_13_O_2_: 237.0921; found: 237.0916.

Ethyl (E)-3-oxo-3-(2-(2-phenylprop-1-en-1-yl)phenyl)propanoate (**6**)

Step A: A solution of stilbene carboxylic acid **5** (4.17 mmol, 994 mg) and 1,1′-carbonyldiimidazole (6.67 mmol, 1.08 g) in dry THF (15 mL) was stirred at room temperature for 12 h.

Step B: To a suspension of potassium ethyl malonate (8.34 mmol, 1.42 g), dry acetonitrile (25 mL) and triethylamine (2.1 mL), magnesium chloride (12.5 mmol, 1.19 g) was added while maintaining the temperature at 20 °C. The reaction mixture was stirred at room temperature for 4 h and then cooled in an ice bath. The solution from step A was added slowly and the resulting mixture was stirred for 12 h. The solvent was removed under vacuum, the residue was taken up in toluene (20 mL), cooled in an ice bath, and aqueous HCl (12%, 10 mL) was slowly added. The mixture was extracted with ethyl acetate. The combined organic layers were washed with aqueous NaHCO_3_, brine, dried over MgSO_4_, filtered and then the solvent was removed under vacuum. The crude product was purified by column chromatography (EtOAc:hexane, 1:12) to give product **6** as an orange oil in 59% yield (2.46 mmol, 760 mg). ^1^H-NMR (250 MHz, CDCl_3_): δ = 8.00–7.30 (m, 10H), 7.12 (s, 0.4H), 5.43 (s, 0.6H), 4.30–4.09 (m, 2H), 2.12 (s, 1.8H), 2.08 (s, 1.2H), 1.37–1.15 (m, 3H) ppm. HRMS (NSI): *m/z* [M + H]^+^ calcd. for C_20_H_21_O_3_: 309.1485; found: 309.1487.

*Ethyl 1-hydroxy-3-methyl-4-phenyl-2-naphthoate* (**7**). β-Keto ester **6** (0.065 mmol, 20 mg) and FeCl_3_ (0.071 mmol, 11 mg) were dissolved in dry CH_2_Cl_2_ (3 mL) under argon at −60 °C and stirred for 5 min. Phenylselenenyl chloride (0.139 mmol, 43 mg) was added to the reaction mixture and stirred while the flask warmed to room temperature; the reaction was then left stirring for 5 h. The mixture was then poured in cold water, extracted with CH_2_Cl_2_ and washed with water. The combined organic layers were dried with MgSO_4_, filtered and concentrated under vacuum. The crude product was purified by preparative TLC (toluene:hexane, 3:2) to give the pure product **7** as a colorless solid in 96% yield (0.062 mmol, 19 mg). ^1^H-NMR (400 MHz, CDCl_3_): δ = 12.59 (s, 1H, OH), 8.46–8.42 (m, 1H), 7.51–7.40 (m, 5H), 7.23–7.16 (m, 3H), 4.49 (q, *J* = 7.1 Hz, 2H), 2.35 (s, 3H), 1.44 (t, *J* = 7.1 Hz, 3H) ppm. ^13^C-NMR (75 MHz, CDCl_3_): δ = 172.9, 161.3, 140.3, 136.0, 132.3, 131.3, 131.0, 129.5, 128.6, 127.1, 126.3, 124.9, 123.9, 123.5, 107.1, 61.9, 21.5, 14.4 ppm. HRMS (NSI): *m/z* [M + H]^+^ calcd. for C_20_H_19_O_3_: 307.1329; found: 307.1332.

## 4. Conclusions

We have demonstrated the successful carbocyclization of a new stilbene derivative using electrophilic selenium reagents in the presence of Lewis acid, to afford the corresponding tetrasubstituted naphthalene derivative in excellent yields. The desired product is obtained through a *6-endo* cyclization, phenylselenium elimination and a 1,2-rearrangement of the phenyl moiety. Although there was the chemical possibility for an alternative rearrangement, the phenyl migration was the only migration observed. The reaction proceeded under very mild reaction conditions and with complete selectivity for the *6-endo* cyclization.
